# Neuroanatomical Correlates of Suicide in Psychosis: The Possible Role of von Economo Neurons

**DOI:** 10.1371/journal.pone.0020936

**Published:** 2011-06-22

**Authors:** Martin Brüne, Andreas Schöbel, Ramona Karau, Pedro M. Faustmann, Rolf Dermietzel, Georg Juckel, Elisabeth Petrasch-Parwez

**Affiliations:** 1 Research Department of Cognitive Neuropsychiatry and Psychiatric Preventive Medicine, University Hospital Bochum, Bochum, Germany; 2 Department of Psychiatry, Ruhr-University of Bochum, University Hospital Bochum, Bochum, Germany; 3 Department of Neuroanatomy and Molecular Brain Research, Institute of Anatomy, Ruhr-University Bochum, Bochum, Germany; Universidad Federal de Santa Catarina, Brazil

## Abstract

Suicide is the most important incident in psychiatric disorders. Psychological pain and empathy to pain involves a neural network that involves the anterior cingulate cortex (ACC) and the anterior insula (AI). At the neuronal level, little is known about how complex emotions such as shame, guilt, self-derogation and social isolation, all of which feature suicidal behavior, are represented in the brain. Based on the observation that the ACC and the AI contain a large spindle-shaped cell type, referred to as von Economo neuron (VEN), which has dramatically increased in density during human evolution, and on growing evidence that VENs play a role in the pathophysiology of various neuropsychiatric disorders, including autism, psychosis and dementia, we examined the density of VENs in the ACC of suicide victims. The density of VENs was determined using cresyl violet-stained sections of the ACC of 39 individuals with psychosis (20 cases with schizophrenia, 19 with bipolar disorder). Nine subjects had died from suicide. Twenty specimen were available from the right, 19 from the left ACC. The density of VENs was significantly greater in the ACC of suicide victims with psychotic disorders compared with psychotic individuals who died from other causes. This effect was restricted to the right ACC. VEN density in the ACC seems to be increased in suicide victims with psychosis. This finding may support the assumption that VEN have a special role in emotion processing and self-evaluation, including negative self-appraisal.

## Introduction

In clinical psychiatry, patient suicide is certainly the most serious incident. Suicidal behavior can be defined as a conscious, intentional act that aims at terminating one's life. Among the functional psychoses, completed suicide occurs in roughly 5% of patients with schizophrenia [1], and similar figures have been published for affective disorders [2]. Although the psychological processes that lead to suicide are heterogeneous in nature, negative self-appraisal, feelings of hopelessness, or social isolation may be particularly relevant [3]. With regard to psychotic disorders patients with good insight into their illnesses have been found to be more prone to commit suicide than patients with poor insight [4]–[5], especially when aware of their negative symptoms and delusional ideation [6]; suicidal behavior can hence be interpreted as the desire to reduce emotional pain.

Functional brain imaging studies into the neuronal representation of psychological pain suggest that the anterior cingulate cortex (ACC) and the anterior part of the insular cortex (AI) are active when subjects evaluate psychologically painful stimuli and when empathizing with others who experience physical pain [7]–[8]. These activation patterns broadly overlap with those implicated in the processing of complex negative emotions such as self-criticism [9], feelings of being socially excluded [10], and in the perception of unfairness [11]. Although the assumption of a common neuronal surrogate of suicidal behavior and empathetic abilities has not been examined systematically, indirect support in favor of such a connection comes from genetic studies. For example, evidence suggests an association of the short allele of the serotonin transporter gene with suicidal behavior [12], especially in the presence of adverse childhood experiences, and the same genetic variation is related to superior recognition of negative emotions in facial expressions [13]. In any event, these issues involve higher-order reflective functioning and social perception, thus rendering them highly significant for the clinical understanding of complex psychopathological conditions [14].

In spite of growing research into the neurobiological correlates of suicidal behavior [15], little is known about the neuronal correlates of the subjective experience of guilt, shame, humiliation, self-derogation, and social exclusion [3], all of which feature suicidal behavior. Here, we explore the hypothesis that von Economo neurons (VENs) may have a specific functional role in the evaluation of these complex emotions, based on the observation that VENs are uniquely located in the brain regions involved, i.e. the rostral ACC, and AI [16].

VENs, named after the neuroanatomist who devoted to them the first systematic description [17], represent a unique spindle-shaped cell type that is mainly located in clusters in layer Vb of the rostral ACC, foremost in Bordmann area (BA) 24, and in the AI of the human brain, with some scattered cell populations in the dorsolateral prefrontal cortex [18]–[20]. While it has long been believed that these cells are specific to apes including humans [21], there is now evidence that VENs also exist in cetaceans [22]–[23] and elephants [24]. In apes both the local numbers and the cell volume of VENs in the ACC have dramatically increased over evolutionary time, with humans reaching the greatest numbers relative to other pyramidal neurons and largest cell soma volumes of VENs compared to other apes [21]. This finding and the convergent evolution of VENs in other highly gregarious animals such as whales and elephants suggest a special role in processing complex social emotions [25]–[26]. This assumption is supported by the observation that the VENs increase dramatically in number during the first years of life, reaching adult figures around 4 years of age [27], around the time when important emotional competencies develop, including empathetic perspective-taking [28]. Moreover, even though the precise functional properties of the VENs are unknown so far [29], it has been demonstrated that these cells express dopamine, serotonin and vasopressin receptors [30], all of which being neurotransmitters that are involved in emotion regulation.

Further evidence for a role of VENs in complex social emotions comes from studies in neurodevelopmental and neurodegenerative disorders. Several studies have shown reduced VEN density or morphological anomalies in individuals with autism [30]–[32], early onset schizophrenia [33], Alzheimer's disease [34], frontotemporal dementia [35]–[36], and agenesis of the corpus callosum [37]. These disorders, though heterogeneous in nature, have in common that affected individuals have profound difficulties in empathizing with others [28], and display underactivations of the ACC and AI in functional brain imaging studies during performance of tasks tapping into empathic abilities [38].

Whether or not VENs can be assigned a role in suicidal behavior is entirely unknown. However, based on the indirect evidence outlined above, we sought to explore this idea by examining the density of VENs in the ACC of subjects with schizophrenia and bipolar disorder who committed suicide compared to individuals with these conditions who died from other (non-violent) causes. Our main hypothesis was that patients who committed suicide would express greater density of VEN in the ACC than subjects who died from natural causes.

## Results

### VEN density in schizophrenia and bipolar disorder

VEN density in patients with schizophrenia (54.45±18.3 VENs/mm^3^) did not differ from that found in post-mortem brains of bipolar patients (56.45±19.46 VENs/mm^3^; t = −.127, df = 37, p = .743).

### VEN density and suicide

Schizophrenia patients who died from suicide (N = 4) had a greater density of VEN in the ACC (76.78±19.8 VENs/mm^3^) compared to suicide victims (N = 5) with bipolar disorder (61.34±18.3 VENs/mm^3^), however, the difference did not reach statistical significance (t = 1.216, df = 7, p = .263). Likewise, the VEN density of non-suicide patients did not differ between schizophrenia (49.48±12.7 VENs/mm^3^) and bipolar disorder subjects (53.66±18.7 VENs/mm^3^; t = −.724, df = 28, p = .475).

As predicted, when pooling the data of suicide versus non-suicide patients, the VEN density was significantly higher in post-mortem brains of suicide victims (68.2±19.5/mm^3^; N = 9) compared to subjects who died from natural causes (51.4±15.6/mm^3^) such as cardiac failure (F = 7.119, df = 1, p = .011). Suicide victims were significantly older (27.9±5.99 years) at first onset of the disorder than non-suicide subjects (20.6±7.44 years), which had no effect on the difference in VEN density between both groups (F = 6.708, df = 1, p = .014). No significant differences between suicide and non-suicide subjects emerged regarding age, brain weight, cortex thickness, thickness of layer Vb and post-mortem fixation interval (all p>.05). However, since the thickness of layer Vb revealed a non-significant, but potentially relevant difference between the suicide and the non-suicide group (F = 3.065, df = 1, p = .089), we introduced the thickness of layer Vb as a covariate in the equation. The difference in VEN density between suicide victims and non-suicide subjects remained highly significant (F = 11.421, df = 1, p = .002). The same was true when entering the cortical thickness as covariate (F = 6.974, df = 1, p = .012). To determine the presence or absence of a rostro-caudal gradient, a repeated-measures ANOVA using VEN density in the four sections examined as within-subject factor and suicide as between-subject factor was carried out, which revealed no effect of the within-subject factor (F = 1.313, df = 3, p = .275), and no interaction of VEN density by group (F = .753, df = 3, p = .506), suggesting that the absence of a rostro-caudal gradient was similar in both the suicide and the non-suicide groups.

**Figure 1:**
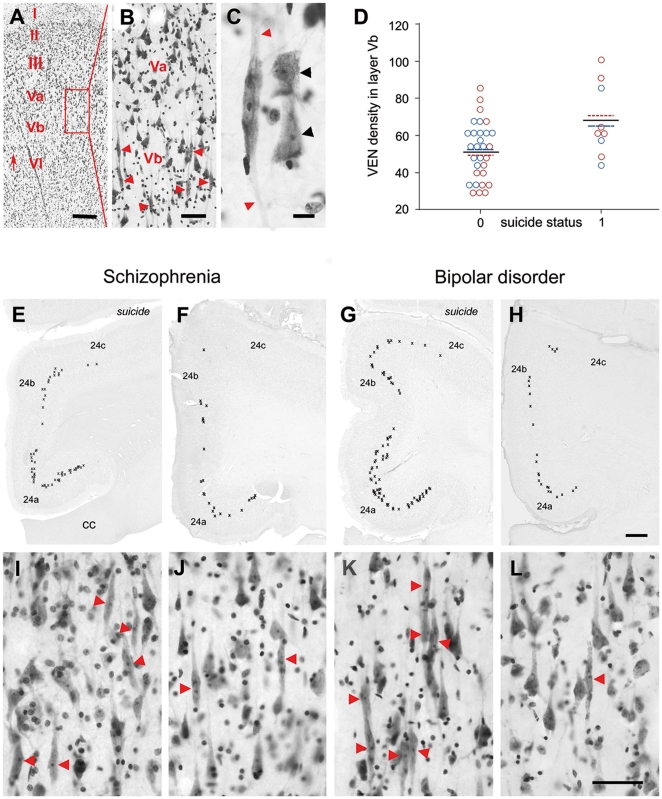
The localization and distribution of VENs in the ACC of suicide victims and non-suicide subjects. (A) Cresyl violet stained section displays the cortical layers I, II, III, Va, Vb and VI of the anterior cingulate cortex (ACC). (B) Numerous von Economo neurons (VENs, arrowheads) are localized in layer Vb, adjacent to layer Va, where large pyramidal cells are frequent. (C) The VENs (for localization see arrow in A) with elongated somata and bipolar dendrites (red arrowheads) can easily be distinguished from neighbored triangular-shaped pyramidal cell somata (black arrowheads). (D) VEN density in the ACC of suicide victims and non-suicide subjects with psychosis (values for the right ACC are shown in red, values for the left ACC are marked in blue; black: total values; “0” indicates no suicide, “1” indicates suicide cases). The representative distributions of VENs in subjects with schizophrenia (E, F) and bipolar disorder (G, H) reflect the high VEN density in suicide victims (E, G) when compared with non-suicide subject (F, H). Suicide victims exhibit more VENs (red arrowheads) clustering (I, K); in non-suicide subjects VENs are localized more solitary (J, K). cc, corpus callosum. Scale bars in A = 2 mm; in B = 50 µm; in C = 10 µm; in H for E–H = 1 mm, in L for I–L = 50 µm.

Notably, when looking separately at the right (N = 6) and left (N = 3) ACC, differences in VEN density were significant only in the right hemisphere (70.23±20.74/mm^3^ VENs in suicide victims versus 48.85±19.74/mm^3^ in non-suicide subjects; F = 4.791, df = 1, p = .042), whereas the difference in VEN density in the left ACC was non-significant (64.13±20.17/mm^3^ VENs in suicide victims versus 53.68±11.09/mm^3^ in non-suicide subjects; F = 1.764, df = 1, p = .202) (Figure 1D).

Interestingly, insight into the psychotic illness did not differ between suicide and non-suicide subjects (F = .587, df = 1, p = .449). However, when looking at the right ACC alone (N = 6), subjects who committed suicide had more insight into the disorder than non-suicide subjects, a finding that reached statistical significance at a trend level (F = 3.777, df = 1, p = .068). No significant differences between right and left hemispheric samples emerged regarding age, sex, brain weight, age at onset, duration of illness and lifetime antipsychotic drug treatment (all p>.05).

### Distribution and morphology of VEN

Since VEN are unevenly distributed across different sections of the rostral ACC and often occur in clusters, we compared the distribution patterns of VENs in BA 24a, BA 24b and BA 24c in brains of suicide victims with non-suicide subject (Fig. 1 E, F, G, H). The suicide brains of both groups (Fig. 1 E, G) showed a higher density than the non-suicides (Fig. 1F, H). The lowest density was detected in layer BA 24c according to reported observations in healthy control cases [15], [32], [37]. Interestingly, in the ACC of non-suicide subjects with lower density, the VENs were especially rare in BA 24b and BA 24c (Fig. 1F, H). Due to the higher density, the VENs showed more clustering in suicide victims (Fig. 1I, K), as compared with the respective area of non-suicide subjects (Fig. 1J, L). The characteristic morphological phenotypes of the VEN with smooth and slender somata and bipolar dendrites could be identified in all groups similar to those described in non-neurological subjects [15], [33]. The dendritic processes may be curved; prominent corkscrewing as described in frontotemporal dementia and Pick's disease [33] or VEN with atypical morphology were rarely observed.

## Discussion

The present study sought to determine the density of VENs in the ACC of patients with psychosis who died of suicide. Our study hypothesis was that suicide victims may have greater VEN density in the ACC, based on the putative role of VENs in processing complex emotions, including empathy, fairness, and more self-centered emotions including negative ones such as guilt, shame, hopelessness, and social exclusion [7], [8], [9], [10], [11], [16].

In line with predictions, suicide victims with psychotic disorders had greater local densities of VENs in the ACC than subjects who died from non-violent causes. The difference in VEN density between suicide victims and non-suicide patients was, to a limited degree, confounded by age at onset of the disorder. Patients who died from suicide were significantly older at age of onset of the disorder than non-suicide patients, which could suggest that the suicide group belonged to a different subtype of psychosis compared to the non-suicide patient group. However, when taking age as covariate into account, the difference in VEN density between the suicide and the non-suicide groups remained significant, suggesting that clinical subtype was not a main factor accounting for the differences in VEN density.

Instead, our finding is, cautiously interpreted, compatible with the assumption that subjects suffering from psychosis are perhaps at greater risk of committing suicide, if their ability to process complex emotions such as painful experiences and empathy is preserved. Put differently, the ability to reflect upon oneself in ways that lead to negative self-appraisal, self-derogation and feelings of shame, guilt and hopelessness may put patients with psychotic disorders at risk of committing suicide [3]. In fact, psychotic patients with good insight into their psychotic illnesses have been found to be more prone to commit suicide than patients with poor insight [5], especially when aware of their negative symptoms and delusional ideation [6]. In accordance with the hypothesis that complex emotions and self-awareness are functionally subserved by VEN activity, the greater VEN density in suicide victims was particularly evident in the right ACC, which is in line with a right hemispheric advantage in processing of negative emotions [40]. Furthermore, we found, at trend level, that suicide victims with a greater VEN density in the right ACC also had higher insight scores than subjects with lower VEN density. Consistent with this observation, insight into psychosis has been identified as a potential risk factor for suicide attempts, particularly, if associated with subjectively poor quality of life and reduced self-esteem [5]. Further support for the interpretation of our findings comes from a recent study of the expression of proteins involved in immune responses. Stimpson and colleagues [41] demonstrated that human VENs are more immunoreactive to an activating-transcription factor (ATF3), interleukin 4 receptor (IL4R-alpha), and neuromedin B (NMB) compared to VENs of other apes, and that this reaction was largely specific for VENs compared to other neurons located in layer Vb of the ACC. In light of the role of these proteins in regulating pain sensitivity, inflammatory reactions, and gut peristalsis, it is well conceivable, though speculative, that they also modulate more complex processes such as psychological pain, empathy to pain, and “gut feelings” [41]. In addition, VENs have been found to be rich in vasopressin, dopamine and serotonin receptors, and these neurotransmitters are known to be critically involved in the regulation of reward and affiliative emotions [30], a finding that may help elucidate the role of genetic variation of neurotransmitter transporters in suicide [12], [13].

The present study has several limitations. First, as regards the association with insight it has to be kept in mind that the number of suicide victims of whom specimen from the right ACC were available was just six individuals, which precludes drawing firm conclusions due to the risk of type-I error. In addition, the method of estimating insight was highly subjective and did not involve standardized rating. Second, we did not calculate the ratio of VENs to the number of pyramidal neurons, a method that could potentially improve accuracy of findings [34]. However, since VENs are unevenly distributed in the ACC and occur in clusters, ratios may have obscured the density in the area investigated. Thus, in accordance with our own previous study [33], we chose to control for tissue shrinking by calculating the VEN density in relation to cortical volume. Furthermore, we took into account the rostro-caudal gradient of VEN density, which did not differ between the suicide and non-suicide groups. A third limitation of our study concerns the fact that correlational evidence between VEN density and suicidal behaviour does not necessarily imply causality. Finally, cell density alone tells little about function; thus, it cannot be ruled out that the VENs of patients with schizophrenia and bipolar disorder display microstructural abnormalities or dysfunctional connectivity with other brain areas.

Our findings are consistent with the hypothesis first put forth by Allman and colleagues [30] that VENs may be assigned a specific role in social cognition. VENs, it seems, are part of a neural circuitry subserving the highest cognitive functions that emerged during human evolution, which entails, perhaps inevitably, the potential of suicidal behavior.

Future studies on the role of VEN in psychopathological conditions and individual symptoms may greatly benefit from more precise clinical data, as well as from exploring VEN density in the FI [32]. Moreover, characterization of neurotransmitter receptors and genes involved in neurogenesis may also help solving the enigmatic role of VEN in normal and abnormal psychology [27].

## Materials and Methods

The Stanley Medical Research Institute reassures that in all cases the next of kin gave permission in writing to examine the brains for research purposes. A detailed statement about the Stanley Brain Collection can be obtained from the Institute's website at www.stanleyresearch.org. Herein, the Stanley Medical Research Institute declares that it “has never knowingly obtained any donation of brain or other tissue without the full consent of available next to kin” and that “information on the Stanley Medical Research Institute and its research was offered to the next of kin at the time of the donation and was provided afterwards upon request.” The study was also approved by the Ethics Committee of the Medical Faculty of the Ruhr-University Bochum, Germany.

Post-mortem ACC brain tissue of 39 individuals with psychotic disorders was obtained from the Stanley Foundation Neuropathology Consortium (SFNC, Chevy Chase, USA). Twenty individuals had a clinical diagnosis of schizophrenia (mean age 45 years; 13 males, seven females), four of whom had committed suicide. Nineteen subjects had bipolar disorder (mean age 47 years; eight male, 11 females), five of whom died of suicide. Twenty specimen were available from the right, 19 from the left ACC. Six of the nine specimen from suicide victims were from the right ACC, three from the left ACC. Details of demographic data and comparisons of relevant tissue variables are presented in Table 1.

**Table 1:** Comparison of demographic and brain related variables between subjects with psychosis who committed suicide and subjects who died from non-suicidal causes.

Variable	*Suicide*	*no Suicide*	*p-value*
***Age (yrs.)***	43.0+/−8.12	47.0+/−9.16	.224, n.s.
***M ∶ F ratio***	17 ∶ 13	4 ∶ 5	.706, n.s.
***Age at onset***	27.9+/−5.99	20.6+/−7.44	.008
***Brain weight (grams)***	1,431.1+/−139.2	1,383.3+/−135.7	.343, n.s.
***Post-mortem interval (hours)***	36.3+/−18.6	33.3+/−15.4	.612, n.s.
***Average density of VENs per mm^3^***	68.2+/−19.5	51.4+/−15.6	.011

Insight was retrospectively estimated as 1 = poor, 2 = fair, or 3 = good, based on individual medical records or reports from the family about whether the individual voluntarily sought treatment, verbalized an understanding of the illness, and complied with medication. Ratings were carried out blind to the results of VEN density.

We examined four frontal 60 µm cryosections of the ACC per brain, each of which located at the same level in each individual according to the section number by the SFNC, as described in [33]. All sections investigated were 2.5 mm apart from one another, with the most rostral full-face section available just behind the genu of the corpus callosum, where VENs are more frequent than at posterior levels [34]. The subareas of the ACC 24a, b and c were identified by cytoarchitectonic criteria [39]. Within these areas layer Vb was outlined as the region of interest (ROI) for VEN counting by using low power (20×) magnification. Layer Vb could be identified in the cresyl violet stained sections adjacent to the layer Va, which is mainly characterized by triangular-shaped pyramidal cells (Fig. 1A; B). Throughout the entire delineated ROI, all VENs were counted in every section investigated at 200× magnification, all target cells had to be localized within the delineated ROI. The VEN could easily be distinguished from the triangular-shaped large pyramidal cells at higher magnification (Fig. 1B, C). Although VENs may vary in morphology, they had to fulfill strict criteria for counting [17], [29], [31], [35]. The elongated somata (Fig. 1B) were always orientated perpendicular to the pial surface. All VEN counted had a distinct nucleus, a visible nucleolus and exhibited the characteristic bipolar dendritic orientation with a single apical and a single basal dendrite (Fig. 1C).

Cell density of VENs was calculated by dividing the total number in each section by the volume (delineated counting area in mm^2^×60 µm section thickness). The density of VENs obtained in all four sections was back-calculated per unit area (1 mm^3^) and used for statistical analysis, which was performed using the Statistical Package for the Social Sciences (SPSS) version 17 for windows. The section thickness was measured at several locations (BA 24 a, b and c) with a calibrated microscope stage (MicroBrightField). The variation between the sections of and between the groups was around ±3 µm.

In order to assess differences in atrophy of the investigated brain tissue, we measured the cortical thickness (layer I–VI) of the ACC in all sections used for counting by outlining the ACC areas at low power (10fold) magnification. To control the rostro-caudal gradient of VEN density, we compared the density of all four sections between the groups.

Normal distribution of the data was indicated by Kolmogorov-Smirnov tests. Between-group differences were examined using univariate ANOVA or student's t-test (two-tailed). P-values smaller than 0.05 were considered significant.

Cell counting and quantitative analysis were performed by two independent raters (R.K.; A.S.) both of whom were blind to the diagnosis. In order to control the concordance of both raters, four sections of 10 subjects counted by A.S. were also counted by R.K. The interrater-reliability was estimated by using the Spearman rank correlation coefficient for the paired results, which was highly significant (r = 0.9273; p = 0.0003). To exclude a systematic error of correlation, the concordance for each of the paired results of the two observers was determined in percentage. The concordance between A.S. and R.K. was 96.4% (range 90.6% to 100%).
